# T Regulatory Cells From Non-obese Diabetic Mice Show Low Responsiveness to IL-2 Stimulation and Exhibit Differential Expression of Anergy-Related and Ubiquitination Factors

**DOI:** 10.3389/fimmu.2019.02665

**Published:** 2019-11-25

**Authors:** Gloria J. Godoy, Carolina Olivera, Daniela A. Paira, Florencia C. Salazar, Yamile Ana, Cinthia C. Stempin, Ruben D. Motrich, Virginia E. Rivero

**Affiliations:** ^1^Centro de Investigaciones en Bioquímica Clínica e Inmunología, CONICET, Córdoba, Argentina; ^2^Departamento de Bioquímica Clínica, Facultad de Ciencias Químicas, Universidad Nacional de Córdoba, Córdoba, Argentina

**Keywords:** NOD mice, regulatory T cells, IL-2 signaling, GRAIL, STUB1, USP7

## Abstract

Foxp3+ Regulatory T cells (Tregs) are pivotal for the maintenance of tolerance. Alterations in their number and/or function have been proposed to occur in the autoimmune-prone non-obese diabetic (NOD) mouse. Comparing the frequencies and absolute numbers of CD4+Foxp3+CD25+ Tregs among 4 to 6-week old NOD, B6, and BALB/c mice, we observed differences in counts and Foxp3 expression in Tregs from secondary lymphoid organs, but not in the thymus. Upon TCR and IL-2 stimulation, NOD Tregs showed lower responses than Tregs from B6 and BALB/c mice. Indeed, NOD Tregs responded with less proliferation and with smaller increments in the expression of CD25, LAP-1, CD39, PD-1, PD-L1, and LAG-3, when *in vitro* cultured for 3 days with anti-CD3/CD28 in the absence or presence of IL-2, Tregs from NOD mice showed to be highly dependent on IL-2 to maintain Foxp3 expression. Moreover, NOD Tregs become producers of IL-17 and INF-gamma more easily than Tregs from the other strains. In addition, NOD Tregs showed lower responsiveness to IL-2, with significantly reduced levels of pSTAT5, even at high IL-2 doses, with respect to B6 and BALB/c Tregs. Interestingly, NOD Tregs exhibit differences in the expression of SOCS3, GRAIL, and OTUB1 when compared with Tregs from B6 and BALB/c mice. Both, at steady state conditions and also after activation, Tregs from NOD mice showed increased levels of OTUB1 and low levels of GRAIL. In addition, NOD Tregs had differences in the expression of ubiquitin related molecules that play a role in the maintenance of Foxp3 cellular pools. Indeed, significantly higher STUB1/USP7 ratios were detected in NOD Tregs, both at basal conditions and after stimulation, compared to in B6 and BALB/c Tregs. Moreover, the addition of a proteasome inhibitor to cell cultures, conferred NOD Tregs the ability to retain Foxp3 expression. Herein, we provide evidence indicating a differential expression of SOCS3, GRAIL, and STUB1/USP7 in Tregs from NOD mice, factors known to be involved in IL-2R signaling and to affect Foxp3 stability. These findings add to the current knowledge of the immunobiology of Tregs and may be related to the known insufficiency of Tregs from NOD mice to maintain self-tolerance.

## Introduction

The autoimmune prone non-obese diabetic (NOD) mouse spontaneously develops type 1 diabetes and also thyroiditis, sialadenitis, and prostatitis with age, therefore serving as an experimental model to study the mechanisms involved in self-tolerance breakdown (1). It is well-known that regulatory T cells that express Foxp3 (Tregs) are pivotal to the maintenance of tolerance and that a failure in its differentiation results in fatal autoimmune lymphoproliferative disease (2, 3). In addition, a wide range of genetic manipulations that impair Treg cell function or end in decreased Tregs numbers also result in increased autoimmunity (2, 3). These data have led to the hypothesis of a possible failure in the numbers and/or functionality of Treg cell populations in NOD mice (4–8). Although many researchers tried to answer this interrogation, current evidence remains controversial (4–8). Some authors studied the generation and function of Tregs showing reduced numbers in NOD mice, with data not corroborated by others who demonstrated increased or similar Tregs numbers (4–8). In addition, several reports show that the suppressor activity of Tregs declined with age in the NOD strain (7). However, a comparative analysis between NOD and C57BL/6 (B6) mice showed that Tregs were equally functional in both strains (8).

It is well-known that Foxp3, together with other transcription regulators, induces Tregs development in the thymus (9). T cell receptor and interleukin-2 receptor-derived signals induce *foxp3* gene expression in developing Tregs (10, 11). IL-2 receptor ligation induces Foxp3 regulation through the binding of STAT5 to the *foxp3* promoter and to a specifically demethylated region known as Conserved Non-coding Sequence-2 (CNS2). The CNS2 region is required for the maintenance of the Foxp3 protein expression and stability in Tregs, but not for the initiation of Foxp3 mRNA transcription (12, 13). Recently, it has been demonstrated that Foxp3 maintenance is also affected by inflammatory cytokines and other factors, which alter post-translational modifications such as ubiquitination, acetylation, and phosphorylation thus regulating the stability of the cellular pools of Foxp3 (14). Indeed, Foxp3 stability has been linked to the activities of the deubiquitinase USP7 and ubiquitinase STUB1, which respectively avoid or promote Foxp3 proteasome degradation (15, 16). Thus, in addition to the transcriptional control of *foxp3* expression, other mechanisms of regulation contribute to the overall abundance and activity of Foxp3, affecting the functions of Tregs and therefore the maintenance of self-tolerance.

The interleukin-2 (IL-2) receptor signaling pathway has been strongly implicated in type 1 diabetes (T1D) susceptibility and also in other autoimmune diseases (1, 17). Polymorphisms in *IL-2, IL-2R*α, and *IL-2R*β are genetic risk factors for several autoimmune diseases (1, 17–19). Genomic studies revealed a significant association between the region encoding *IL-2* with T1D, celiac disease, rheumatoid arthritis, autoimmune thyroid diseases and multiple sclerosis (17–22). Moreover, NOD mice also showed a strong genetic susceptibility for autoimmune diabetes mapped to the chromosome 3 region encompassing the *IL-2-IL-21* gene (region *Idd3*) (1, 23). Mice possessing susceptibility alleles at *Idd3* have reduced IL-2 levels in comparison to mice with B6-derived alleles. Furthermore, *Idd3* alleles also affect the development of other autoimmune diseases in NOD mice such as Sjogren's syndrome manifestations, experimental autoimmune encephalitis and others (1, 23, 24).

It is well-known that IL-2 binding to the IL-2R activates associated JAK1 and consequently STAT5, and, that activated STAT5 binds to CNS2 favoring Treg cell stability (25). Once activated, STAT5 dimerizes and translocates into the nucleus where it initiates the transcription of different genes, including negative regulators such as SOCS3. In turn, SOCS3 feeds back into the signaling cascade desensitizing the IL-2R by inactivating pJAK1 (26). On the other hand, the ubiquitin- ligase named “gene related to anergy in lymphocytes” or GRAIL is well-known as an E3 ubiquitin-protein ligase that participates in anergy signaling by limiting activation induced by IL-2 (27, 28). Moreover, GRAIL is regulated by Otubain-1 (OTUB1), a deubiquitinating enzyme that acts as a destabilizing GRAIL protein (29). Interestingly, it has been reported that Tregs express 10 times more GRAIL mRNA than conventional T cells and that GRAIL deficient Tregs exhibited reduced suppressive function (30). However, it has not yet been investigated if Tregs from mice with different susceptibility to autoimmunity have differential expression of these molecules.

In the present report, by comparing Tregs from the autoimmune prone NOD mice with B6 and BALB/c mice, we provide evidence showing that NOD mice exhibit differences in Treg cell counts and of their ability to be stimulated *via* TCR plus IL-2. Our results also demonstrate that Tregs from NOD mice express reduced and higher levels of GRAIL and SOCS3, respectively, as well as increased expression of ubiquitinases known to favor Foxp3 proteasome degradation. Altogether, that results in a pool of Foxp3^+^ Tregs insufficient to maintain self-tolerance.

## Materials and Methods

### Mice

Female, non-diabetic, 4 to 6-week old mice were used in all the experiments. NOD, B6, and BALB/c mice were purchased from Jackson Laboratory and then bred and maintained under specific pathogen-free conditions in the animal facility of the Centro de Investigaciones en Bioquímica Clínica e Inmunología, Universidad Nacional de Cordoba, Argentina. Animals were maintained in a 16 h light−8 h dark cycle, at 20 ± 2°C, with food and water *ad libitum*. All experiments were approved by and conducted in accordance with the guidelines of the Committee for Animal Care and Use of the Facultad de Ciencias Químicas, Universidad Nacional de Córdoba (Res. HCD 240/216), in strict accordance with the recommendation of the Guide for the Care and Use of Laboratory Animals of the NIH (NIH publication 86-23). Animals were not subjected to any treatment. All efforts were made to minimize suffering and discomfort.

### Flow Cytometry

Single cell suspensions were prepared from spleen and lymph nodes (LN) of individual mice and stained for surface markers. Dead cells were excluded using Live-dead fixable (Invitrogen). The following antibodies were used: CD4, CD25 (PC61), GITR, CD73, CD39, CD3, CD8, LAG-3, LAP-1, PD-1, PD-L1, pSTAT5, GRAIL (Abcam, Cambridge, UK), OTUB-1 (Abcam), Ki67, Bcl-2, STUB-1 (Abcam), and USP7 (Abcam). For intracellular Foxp3, CTLA-4, and Ki67 staining a Foxp3 Staining Kit (eBioscience) was used. For the assessment of intracellular GRAIL, OTUB-1, STUB-1, and USP7 expression, cells were first stained with surface antibodies and then fixed and permeabilized with the Foxp3 Staining Kit (eBioscience, San Diego, California, USA) for 30 min followed by reacting with rabbit anti-GRAIL, anti-OTUB-1, anti-STUB-1, and anti-USP7 primary Ab (Abcam) for 45 min. After washing, cells were stained for 20 min with FITC or Alexa Fluor 647-anti-rabbit IgG (Biolegend, San Diego, California, USA). Finally, cells were washed twice with a saline solution of 2% FBS and stored at 4°C in the dark until analysis. To measure cytokine production, cells were activated for 4 h with PMA (50 ng/ml; Sigma-Aldrich), ionomycin (500 ng/ml; Sigma-Aldrich), and brefeldin A (2 μM; eBioscience) in 96-well U-bottom plates. After cell staining for surface molecules, cells were fixed and permeabilized with Staining Set (eBioscience) and stained with anti-mouse IFN-γ and IL-17 mAb (BD Biosciences) according to the manufacturer's instructions. For phospho-STAT5 detection, cells were fixed with (2% wt/vol) paraformaldehyde for 20 min at room temperature, permeabilized with 100% (vol/vol) methanol for 10 min on ice. After extensive washing, cells were stained with anti-pStat5 (Tyr-694) (eBiosciences). Data were collected on FACS-CANTO II or LSRFortessa flow cytometers (BD Biosciences, San Jose, CA, USA) and analyzed using FlowJo software (version 7.6.2). Proper compensation using Fluorescence Minus One (FMO) controls were used.

### Cell Purification and Sorting

Cells were obtained from pooled spleens freshly collected by mechanical disruption. Mononuclear cell suspensions (MNCs) were prepared under sterile conditions in HBSS (Sigma-Aldrich, St. Louis, MO, USA) and separated on Ficoll-Hypaque PREMIUM 1.084 (GE Healthcare Bio-Sciences AB, Uppsala, Sweden) centrifugation gradients. CD4^+^ cells were enriched from MNCs using EasySep™ Mouse CD4^+^ T Cell Isolation Kit (StemCel Technologies, Vancouver, Canada). Finally, CD4^+^CD25^hi^ cells (Treg) were sorted from CD4^+^ cells enriched with a FACSAria (BD Biosciences, San Diego, CA, USA) on the basis of CD4 and CD25 expression, ensuring purity greater than 97%. FoxP3 expression was evaluated by intracellular staining with the FJK-16s mAb (eBioscience).

### *In vitro* Treg Cell Activation

In most experiments, CD4^+^CD25^+^ purified T cells were cultured at a concentration of 0.5 × 10^6^ cells/ml per well in triplicate for each condition and resuspended in RPMI 1640 (Life Technologies, Carlsbad, CA, USA) supplemented with 10% FBS (Gibco, Waltham, MA, USA), 2 mM L-glutamine, 0.1% gentamicin, and 50 mM β-Mercaptoethanol. Plates were incubated with plate-bound anti-CD3/CD28 (1 and 0.25 μg/ml, respectively) (BD Biosciences) and different amounts of rIL-2 (Peprotech, Rocky Hill, NJ, USA) as indicated for 72 h at 37°C, 5%CO_2_ incubator. In some experiments the proteasome inhibitor MG-132 (Sigma-Aldrich) was added at a concentration of 5 μM during the last 12 h of the culture.

### RT-PCR Assay

Total RNA was isolated from 3.10^6^ sorted CD4^+^CD25^hi^ splenic Treg cells (12 mice per mouse strain) using TRizol reagent (Invitrogen, Carlsbad, CA, USA) and reverse-transcribed into cDNA by using Reverse Transcription System (Promega, Madison. WI, USA). cDNA was amplified by real time PCR with the predesigned TaqMan gene expression assays and reagents (Applied Biosystems, Foster City, CA, USA), according to the manufacturer's instructions. Probes with the following Applied Biosystems assay identification numbers were used: Rnf128, Mn00480990.m1 and Rn18s, Mn03928990.g1 (31). For each sample, mRNA abundance was normalized to the amount of 18S RNA and expressed as arbitrary units. The primers used for amplification of *OTUB1, SOCS3, STUB1, and USP7* genes were: *OTUB1_*F: 5′-CGACTCCGAAGGTGTTAACTG T-3′, and *OTUB1*_R: 5′-GAG GTCCTT GAT CTT CTG TTG G-3′ (Macrogen, Seoul, Korea), *SOCS3*_F: 5′-TGC GCC TCA AGA CCT TCA G-3′, and *SOCS3*_R: 5′-GCT CCA GTA GAA TCC GCT CTC-3′ (Macrogen); *STUB1*_F: 5′-CCC CCG CCC CTC CCG CAC TC-3′, and *STUB1*_R: 5′-CAT GCC AGC TCC GCC CCA CA-3′ (Macrogen); *USP7*_F: 5′-CCC TCC GTG TTT TGT GCG A-3′, and *USP7*_R: 5′-GAC CAT GAC GTG GSS TCA GA-3′ (Macrogen). The primers used for normalization were *eef2* housekeeping gene as follows: F: 5′-AAG CTG ATC GAG AAG CTG GA-3′, and R: 5′-CCC CTC GTA TAG CAG CTC AC-3′ (Sigma-Aldrich). Quantitative real time PCR was performed on a StepOne^TM^ instrument (Life Technologies) using SYBR^®^ Select Master Mix (Life Technologies). Each qPCR experiment was performed at least in triplicates, in a final volume of 20 μl. After performing thermal cycling, qPCR amplification data were analyzed using StepOne software (Applied Biosystems).

### Statistical Analysis

Statistical analysis was performed using one or two-way ANOVA with Bonferroni *post-hoc* test analysis. Data are shown as mean ± SEM in the graphs. Statistical tests were performed using the GraphPad Prism 7.0 software (GraphPad Software Inc., La Jolla, CA, USA). *P-*values ^*^ < 0.05, ^**^ < 0.01, ^***^ < 0.001, and ^****^ < 0.0001 were considered significant in all analyses.

## Results

### Reduced Treg Cell Counts and Tregs Foxp3/CD25 Expression in NOD Mice

**Figure 1:**
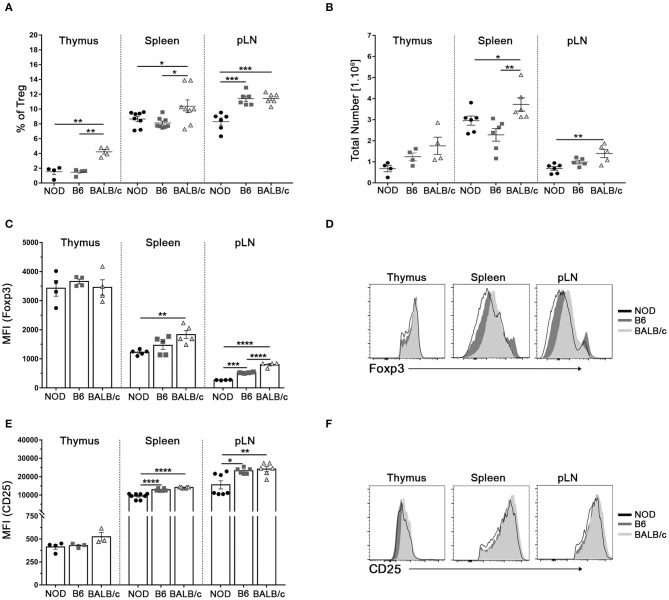
Reduced Treg cell counts and Tregs Foxp3/CD25 expression in NOD mice. **(A)** Scatter plots show the frequency of Foxp3^+^CD4^+^CD25^+^ (Treg) cells in the CD4^+^ live cell population of the thymus, spleen, and a pool of inguinal and popliteal lymph nodes (pLN) from 4 to 6-week old NOD, B6, and BALB/c mice. **(B)** Absolute numbers of Treg cells in the thymus, spleen, and pLN from NOD, B6, and BALB/c mice. **(C)** Bar graph showing Mean Fluorescence Intensity (MFI) values for Foxp3 in Tregs from the thymus, spleen, and pLN from NOD, B6, and BALB/c mice. **(D)** Representative histograms of Foxp3 expression in Tregs from the thymus, spleen, and pLN in all strains under study. **(E)** Bar graph showing MFI values for CD25 in Tregs from the thymus, spleen, and pLN from NOD, B6, and BALB/c mice. **(F)** Representative histograms of CD25 expression on CD4^+^ cells from the thymus, spleen, and pLN in all the mouse strains under study. Gates were performed on viable cells using Live-dead fixable (Invitrogen) dye and Fluorescence Minus One (FMO) controls. Data are shown as mean ± SEM, *n* = 4–8 mice per group, and are representative of three independent experiments with essentially the same results. The *p-*values were obtained using one-way ANOVA followed by Bonferroni *post-hoc* analysis as appropriate. ^*^p < 0.05, ^**^p < 0.01, ^***^p < 0.001, and ^****^p < 0.0001.

Variable data have been reported on the proportion of Tregs in the autoimmune prone NOD mice with respect to B6 mice and other mouse strains (4, 6). We first analyzed the frequencies of CD4^+^Foxp3^+^CD25^+^Tregs among NOD, B6, and BALB/c mice from our animal facility colony. All mice analyzed were young (4 to 6-week old) to ensure they were neither pre diabetic nor have T-cell infiltration in pancreas or other tissues. Foxp3^+^ cell frequencies and absolute numbers were analyzed within single positive (CD4^+^CD8^−^) thymic cells and CD4^+^ cells from spleen and lymph nodes following gating strategies represented in Supplementary Figure 1. Significantly reduced frequencies of CD4^+^Foxp3^+^ Tregs were observed in thymus and spleen from NOD and B6 mice with respect to BALB/c mice (Figure 1A). In addition, NOD mice showed significantly reduced frequencies of Foxp3^+^ Tregs in pooled inguinal and popliteal lymph nodes (pLN) than B6 and BALB/c mice (Figure 1A). When analyzing absolute counts of CD4^+^Foxp3^+^ Treg cells, decreased numbers were observed in the spleen and pLN (but not in the thymus) from NOD mice with respect to the other mouse strains (Figure 1B). Analyses of the relative expression of Foxp3 on Tregs from different tissues revealed that spleen Tregs from NOD mice expressed significantly lower protein levels when compared with those from BALB/c mice, whereas no differences were observed between Tregs from NOD and B6 mice (Figures 1C,D). Similar results were observed when analyzing Foxp3 expression in pLN Tregs, where more elevated Foxp3 levels were detected in BALB/c Tregs than B6 Tregs, whereas NOD Tregs presented the lowest values. Interestingly, Foxp3 expression did not significantly differ among thymus Tregs from the mouse strains under study, indicating that Tregs egress from the thymus expressing comparable amounts of Foxp3. In accordance, similar levels of CD25 expression were detected in thymus Tregs from the different mouse strains under study, although lower levels were observed in spleen and pLN Tregs from NOD mice with respect to B6 and BALB/c mice (Figures 1E,F). Altogether, these results indicate that NOD mice have a generalized defect in maintaining Treg cell numbers and Foxp3 Treg cell levels in secondary lymphoid organs.

### Tregs From NOD Mice Show a Decreased Response to TCR Plus IL-2 Stimulation

**Figure 2:**
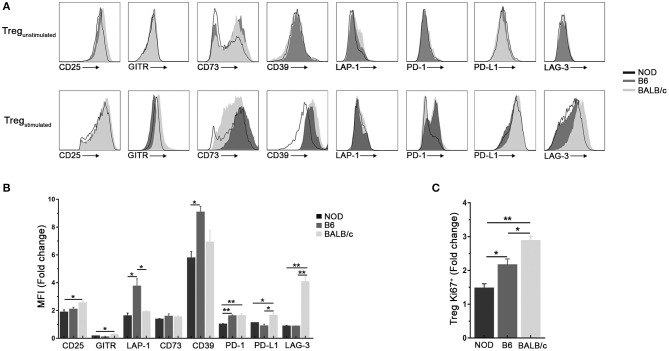
Tregs from NOD mice show low responsiveness to TCR plus IL-2 stimulation. **(A)** Representative histograms of CD25, GIRT, CD73, CD39, LAP-1, PD-1, PD-L1, and LAG-3 expression in purified splenic Treg (CD4^+^CD25^hi^) cells from NOD, B6, and BALB/c mice without stimulation and after αCD3/CD28 plus rIL-2 stimulation (100 UI/ml). **(B)** Bar graph showing values of MFI fold change relative to values found in purified unstimulated Tregs from the mouse strains under study. **(C)** Bar graph showing values of Ki67^+^ Tregs percentage fold change relative to values found in unstimulated Tregs from the mouse strains under study. Gates were performed on viable cells using Live-dead fixable (Invitrogen) dye and Fluorescence Minus One (FMO) controls. To purify Tregs, 12 mice per mouse strain were used. Data correspond to at least four replicates (mean ± SEM) of a single sample for each condition and mouse strain analyzed. The analysis is representative of at least three independent experiments with essentially the same results. The *p-*values were obtained using one-way ANOVA followed by Bonferroni *post-hoc* analysis as appropriate. ^*^p < 0.05 and ^**^p < 0.01.

In classical *in vitro* Tregs function assays, Tregs and T effector cells are placed together so interpretation of results is sometimes difficult (3, 32). Available reported evidence indicates both, greater resistance to suppression of T effector cells and lower suppressor capacity of Tregs from NOD mice (8, 33–35). For that reason, sorted CD4^+^CD25^hi^ T cells (purity > 97%) were *in vitro* stimulated for 3 days to assay functional response of Tregs from the different mouse strains after TCR ligation in the presence of IL-2. Gated CD4^+^Foxp3^+^ cells (Supplementary Figure 2) were assayed for the expression of different molecules related to functional activity. After 3 days of αCD3/CD28 stimulation in the presence of IL-2, an important up regulation of different molecules involved in Treg functional activity was observed in Tregs from all mouse strains under study (Figures 2A,B). Stimulated Tregs showed enhanced expression of CD25, LAP-1, CD73, CD39, PD1, PDL1, and LAG-3. Approximately, a 2-fold increase in the expression of CD25 was detected in stimulated Tregs from NOD and B6 mice with, which was significantly lower to that observed in stimulated Tregs from BALB/c mice (Figures 2A,B). The expression of LAP-1 also showed a 2-fold increase in stimulated Tregs from NOD and BALB/c mice, while Tregs from B6 mice showed a 4-fold increment for this cell marker. CD73 expression showed a similar increment in all mouse strains under study, while GITR expression did not show important increments after stimulation (Figures 2A,B). CD39 expression increased six to seven times in stimulated Tregs cells from NOD and BALB/c mice, while a significantly greater increase was detected in stimulated Treg cells from B6 mice. In the case of PD1 and PD-L1, stimulated Treg cells from NOD mice showed smaller increments when compared with stimulated Tregs from the other mouse strains. Finally, LAG-3 expression showed a 4-fold increase in stimulated Tregs from BALB/c mice, with lower increments in stimulated Tregs from NOD and B6 mice. We also assessed intracellular Ki67 expression as a marker of *in vitro* cell proliferation. Significantly reduced expression of Ki67 was observed in Tregs from NOD mice upon TCR ligation plus IL-2 stimulation when compared with B6 and BALB/c mice (Figure 2C). Altogether, these results show that, after TCR plus IL-2 stimulation, Tregs from NOD mice respond more weakly, with a lower proliferative capacity and decreased expression of molecules involved in regulatory functions.

### Tregs From NOD Mice Show Less *in vitro* Stability in Conditions of Low IL-2 Abundance

**Figure 3:**
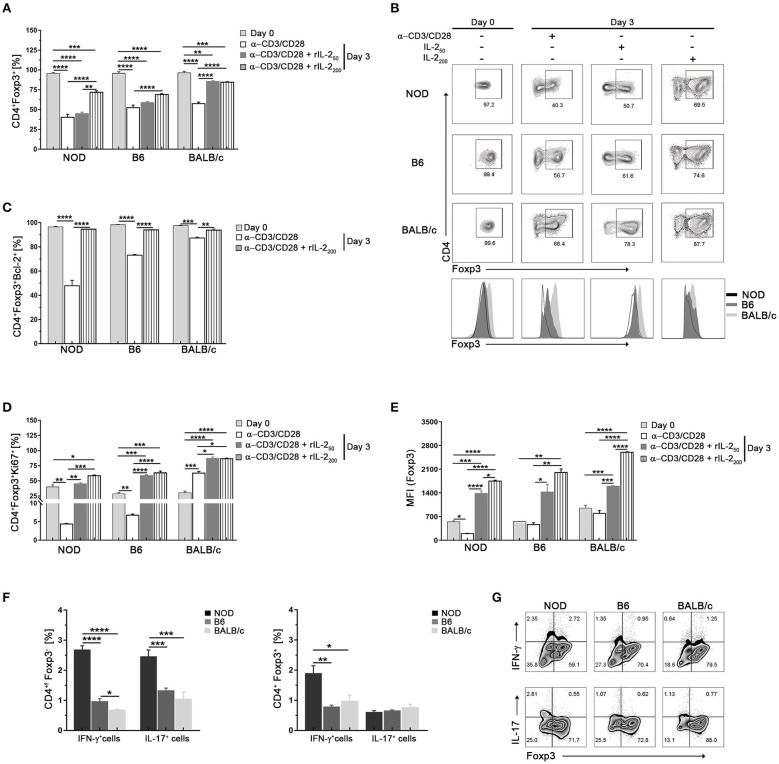
Tregs from NOD mice show less *in vitro* stability in conditions of low IL-2 abundance. **(A)** Bar graph showing the frequencies of Foxp3^+^CD4^+^ cells at days 0 and 3 after stimulation with αCD3/CD28 in the absence or presence of rIL-2 (50–200 UI/ml). **(B)** Representative contour plots and histograms of Foxp3 expression in sorted splenic Treg (CD4^+^CD25^hi^) cells from NOD, B6, and BALB/c mice at day 0 and after 3 days of stimulation with αCD3/CD28 in the absence or presence of rIL-2. **(C)** Bar graph showing the frequency values of CD4^+^Foxp3^+^Bcl-2^+^ cells at days 0 and 3 after stimulation with αCD3/CD28 in the absence or presence of rIL-2 (200 UI/ml). **(D)** Bar graphs showing the frequencies of CD4^+^Foxp3^+^Ki67^+^ cells at days 0 and 3 after stimulation with αCD3/CD28 in the absence or presence of rIL-2 (50–200 UI/ml). **(E)** Bar graph showing MFI values for Foxp3 in CD4^+^Foxp3^+^ cells at days 0 and 3 after stimulation with αCD3/CD28 in the absence or presence of rIL-2 (50–200 UI/ml). **(F)** Bar graphs showing the frequencies values of CD4^+^Foxp3^−^ (non-Tregs) and CD4^+^Foxp3^+^ (Tregs) expressing IL-17^+^ and INF-γ^+^ cells after 3 days of culture with αCD3/CD28 plus rIL-2 (200 UI/ml). **(G)** Representative contour plots showing the frequencies of CD4+Foxp3^+^ and CD4+Foxp3^−^ cells producing IFN-γ and IL-17 after 3 days of culture with αCD3/CD28 plus rIL-2 (200 UI/ml). Gates were performed on viable CD4+ cells using Live-dead fixable (Invitrogen) dye and Fluorescence Minus One (FMO) controls. To purify Tregs, 12 mice per strain were used. Data correspond to at least four replicates (mean ± SEM) of a single sample for each condition and mouse strain analyzed. The analysis is representative of at least three independent experiments with essentially the same results. The *p-*values were obtained using one-way ANOVA followed by Bonferroni *post-hoc* analysis as appropriate. ^*^p < 0.05, ^**^p < 0.01, ^***^p < 0.001, and ^****^p < 0.0001.

We next compared Foxp3 expression in Tregs from the different mouse strains under limiting trophic cytokine conditions, which has been shown to lead to a gradual loss of Foxp3 (36, 37). Sorted CD4^+^CD25^hi^ (>97% CD4^+^Foxp3^+^) were *in vitro* cultured with αCD3/CD28 plate-bound antibodies in the absence or presence of increasing doses of IL-2 (Figure 3). After 3 days of culture with αCD3/CD28 stimulation without IL-2 addition, CD4^+^Foxp3^+^ cell frequencies decreased significantly in cultured Tregs from all mouse strains, but to a minor extent in Tregs from BALB/c mice (Figures 3A,B). In the absence of IL-2 more than 80% of cultured Treg cells from BALB/c mice were Bcl-2^+^ (Figure 3C), and ~60% were able to proliferate (Figure 3D). The opposite pattern was observed in cultured Tregs from NOD mice. The absence of IL-2 caused an important fall in the frequencies of CD4^+^Foxp3^+^ cells (Figures 3A,B) accompanied with the lowest counts of Bcl-2^+^ cells (Figure 3C), and ~5% of proliferating cells (Figure 3D). An intermediate pattern was observed in sorted Tregs from B6 mice, in which 70% of cells were Bcl-2^+^ and a little more than 5% of were proliferating cells. The presence of IL-2 during stimulation allowed the maintenance of Foxp3, Bcl-2, and Ki67 expression in high percentages of cultured Tregs from B6 and BALB/c mice. However, only high IL-2 doses allowed the ability to keep Foxp3 expression in about 70%, and Bcl-2 expression in more than 90% of cultured Tregs from NOD mice, highlighting the crucial importance of IL-2 as a survival factor for Tregs, especially for those derived from NOD mice (Figures 3A–D). When analyzing not only frequencies of positive cells, but also Foxp3 expression levels under experimental conditions, we observed that Tregs from BALB/c mice maintained Foxp3 expression levels under TCR stimulation without IL-2 and significantly increased them in a dose dependent manner when cells were stimulated with the further addition of IL-2 (Figure 3E). Tregs from B6 mice also maintained Foxp3 expression levels under TCR stimulation without IL-2 and after stimulation with increasing IL-2 doses enhanced Foxp3 levels but reaching values lower than those of Tregs from BALB/c mice. Remarkably, NOD Tregs showed a significant loss of Foxp3 expression after stimulation for 3 days with αCD3/CD28 without IL-2. However, enhanced Foxp3 expression was observed when NOD Tregs were stimulated with αCD3/CD28 plus high IL-2 doses, although never reaching the values observed in Tregs from the other mouse strains (Figure 3E). These results indicate that Tregs from NOD mice are strongly dependent of IL-2 to survive and retain Foxp3 expression levels. On the contrary, Tregs from BALB/c mice were the most stable ones under the experimental conditions assayed. Besides being able to maintain Foxp3 expression, Tregs from BALB/c mice also increased more than three times its expression levels after stimulation with IL-2 (Figure 3E).

Using standard expansion protocols (αCD3/CD28 plus IL-2), it has been shown that an important Treg cell expansion can be achieved *in vitro*, but also that a fraction of cells loose Foxp3 expression and start producing IL-17 and IFN-γ (14, 38). Although at day zero IL-17^+^ or INF-γ^+^ cells were no detected (<0.1% for all strains, data not shown) in our setting, after 3 days of culture with αCD3/CD28 plus IL-2 stimulation higher percentages of Foxp3^−^ (*ex*-Treg) and Foxp3^+^ cells also expressing IL-17 or INF-γ were detected in cultured Tregs from NOD mice with respect to the other mouse strains (Figures 3F,G). These data suggest that Tregs from NOD mice have a higher dependence on IL-2 to maintain Foxp3 expression and they lose its expression and become producers of IL-17 and INF-γ more easily than Treg cells from the other mouse strains.

### Tregs From NOD Mice Show Lower Responsiveness to IL-2 Stimulation and Exhibit Differences in the Expression of SOCS3, GRAIL, and OTUB1

**Figure 4:**
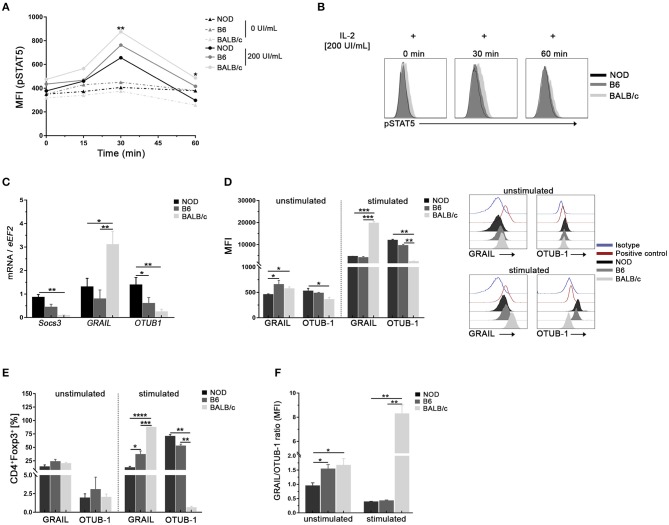
Tregs from NOD mice show low responsiveness to IL-2 stimulation and exhibit differences in the expression of SOCS3, GRAIL, and OTUB1. **(A)** Mean Fluorescence Intensity for pSTAT5 after 0, 30, and 60 min of stimulation with or without rIL-2 (200 UI/ml). Values in the absence of IL-2 for all time-points are represented by dotted lines. **(B)** Representative histograms of pSTAT5 expression in sorted splenic Treg (CD4^+^CD25^hi^) cells from NOD, B6, and BALB/c mice after 0, 30, and 60 min of stimulation with rIL-2 (200 UI/ml). **(C)** Bar graphs of mRNA for SOCS3, GRAIL, and OTUB1 from sorted splenic Tregs from NOD, B6, and BALB/c mice. **(D)** Bar graphs (left) and representative histograms (right) showing MFI for GRAIL and OTUB1 in purified splenic Tregs from NOD, B6, and BALB/c mice, unstimulated or stimulated with rIL-2 during 30 min. **(E)** Bar graphs showing frequencies of GRAIL^+^ and OTUB1^+^ cells in unstimulated and rIL-2 stimulated Tregs from NOD, B6, and BALB/c mice (30 min). **(F)** GRAIL/OTUB1 MIF ratios in unstimulated and stimulated Tregs from NOD, B6, and BALB/c mice. To purify Tregs, 12 mice per strain were used. Data correspond to at least four replicates (mean ± SEM) of a single sample for each condition and mouse strain analyzed. The analysis is representative of at least three independent experiments with essentially the same results. The *p*-values were obtained using one- and two-way ANOVA followed by Bonferroni *post-hoc* analysis as appropriate. ^*^p < 0.05, ^**^p < 0.01, ^***^p < 0.001, and ^****^p < 0.0001.

Since the decreased responsiveness to IL-2 could be due to altered IL-2 receptor signaling, we assayed the phosphorylation status of STAT5 (pSTAT5) in Tregs from the mouse strains under study at different time-points after *in vitro* stimulation with high doses of IL-2 (Figures 4A,B). As shown in Figure 4A, no changes in pSTAT5 levels were observed in the absence of IL-2 for all time-points and samples analyzed (dotted lines). After IL-2 stimulation, increased levels of pSTAT5 were detected in Tregs from all mouse strains reaching peak values at 30 min post stimulus, with the highest and lowest levels observed in Tregs from BALB/c and NOD mice, respectively (Figure 4A). Analysis performed after 60 min of IL-2 stimulation revealed a decline in pSTAT5 levels in Tregs from all the mouse strains under analysis, although maintaining the differences of expression among mouse strains (Figures 4A,B). It is interesting to highlight that even at high doses of IL-2, STAT5 activation in NOD Tregs did not reach the values observed in Tregs from the other mouse strains (Figures 4A,B).

Next, we performed experiments to investigate whether Tregs from NOD mice had differences in the expression of the IL-2 signaling cascade regulators such as SOCS3, a negative regulator of pJAK1 (25). In addition, we analyzed if Tregs from NOD mice had differences in the E3 ubiquitin ligase GRAIL and its regulator OTUB1 (27–29). As shown in Figure 4C, higher levels of SOCS3 and OTUB1 mRNA were detected in purified Tregs from NOD mice with respect to B6 and BALB/c mice. On the contrary, lower levels of GRAIL mRNA were detected in Tregs from NOD and B6 mice (Figure 4C). The analysis of protein expression was performed in unstimulated and stimulated Tregs from all mouse strains (Figure 4D). Lower levels of GRAIL were observed in unstimulated Tregs from NOD mice when compared with B6 and BALB/c mice, whereas the expression of the OTUB1 protein showed the opposite pattern (Figure 4D). After IL-2 stimulation, an important up regulation of GRAIL and OTUB1 was observed in Tregs from all mouse strains under study (Figure 4D). However, NOD and B6 Tregs expressed significantly lower levels of GRAIL and enhanced levels of OTUB1 with respect to BALB/c mice (Figure 4D). The frequencies of GRAIL^+^ or OTUB1^+^ were not different among unstimulated Tregs from mouse strains under study (Figure 4E). However, after stimulation the highest proportion of GRAIL^+^ cells were detected in stimulated Tregs from BALB/c mice, with intermediate values in Tregs from B6 mice and the lowest percentages in Tregs from NOD mice (Figure 4E). On the contrary, higher frequencies of OTUB1^+^ cells were detected in stimulated Tregs from NOD and B6 mice (Figure 4E). When the GRAIL/OTUB1 ratio was analyzed, significantly lower values were observed in NOD Tregs at basal conditions (unstimulated cells, Figure 4F). In addition, GRAIL/OTUB1 ratios after IL-2 stimulation, showed significantly lower values in the NOD and B6 Tregs than in Tregs from BALB/c mice (Figure 4F). Interestingly, GRAIL and OTUB1 expression levels showed no differences between NOD and B6 Tregs (Figure 4D). However, the GRAIL/OTUB1 ratio was higher in unstimulated Tregs from B6 mice with respect to NOD mice, showing again that highly and mildly susceptible mice to develop autoimmunity may share some Tregs features. These data indicate that Tregs from NOD mice show decreased STAT5 activation accompanied with the highest SOCS3 expression and lowest GRAIL/OTUB1 expression ratio.

### Treg From NOD Mice Show Higher Expression of Ubiquitination Related Molecules

**Figure 5:**
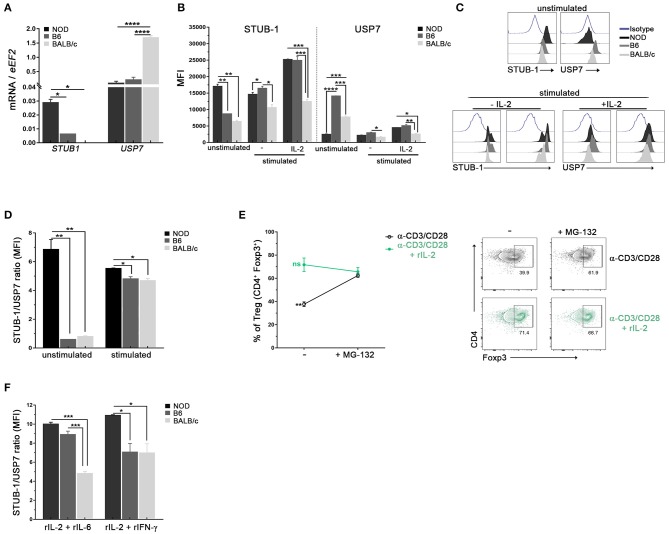
Tregs from NOD mice show high expression of ubiquitination related molecules. **(A)** Bar graphs of mRNA for STUB-1 and USP7 from sorted splenic Treg cells of NOD, B6, and BALB/c mice. **(B)** Bar graphs showing MFI for STUB-1 and USP7 in unstimulated and stimulated purified splenic Treg cells from NOD, B6 and BALB/c mice. **(C)** Representative histograms of STUB-1 and USP7 expression in unstimulated and αCD3/CD28 plus rIL-2 stimulated Treg cells from NOD, B6, and BALB/c mice **(D)** STUB-1/USP7 MFI ratio in unstimulated and stimulated Treg cells from NOD, B6 and BALB/c mice. **(E)** Frequencies (left) and representative contour plots (right) of purified Treg cells *in vitro* stimulated with αCD3/CD28 plus or without rIL-2 for 3 days, and in the absence or presence of the proteasome inhibitor MG-132. The proteasome inhibitor MG-132 was added at a concentration of 5 μM during the last 12 h of the culture. **(F)** STUB-1/USP7 MFI ratios of CD4^+^Foxp3^+^ Treg cells from NOD, B6, and BALB/c mice after 3 days of stimulation with rIL-2 plus rIL-6 or rIFN-γ. To purify Tregs, 12 mice per strain were used. Data correspond to at least four replicates (mean ± SEM) of a single sample for each condition and mouse strain analyzed. The analysis is representative of at least three independent experiments with essentially the same results. The *p-*values were obtained using one-way ANOVA followed by Bonferroni *post-hoc* analysis as appropriate. ^*^p < 0.05, ^**^p < 0.01, ^***^p < 0.001, and ^****^p < 0.0001.

Foxp3 post-translational modifications such as ubiquitination by STUB-1 or deubiquitination by USP7 are known to either deplete or stabilize the cellular pools of Foxp3, respectively (39, 40). Taking that into account, we performed experiments to investigate whether Tregs from NOD mice had differences in the expression of molecules involved in ubiquitination processes in steady state conditions. As can be seen in Figure 5A, higher levels of STUB1 mRNA were detected in purified Tregs from NOD mice with respect to B6 and BALB/c mice. On the contrary, lower levels of USP7 mRNA were detected in Tregs from NOD and B6 mice (Figure 5A). The analysis of protein expression revealed lower levels of USP7 in unstimulated Tregs from NOD mice when compared with Tregs from B6 and BALB/c mice (Figures 5B,C). The opposite pattern was observed for STUB1, which expression was higher in unstimulated Tregs from NOD mice when compared with Tregs from B6 and BALB/c mice (Figures 5B,C). The expression of STUB1 and USP7 was also analyzed after 3 days of culture with αCD3/CD28 plus or without IL-2 stimulation. On the one hand, the highest levels of STUB1 expression were consistently detected in Tregs from NOD and B6 mice cultured with αCD3/CD28 with or without the addition of IL-2 (Figures 5B,C). On the contrary, Tregs from BALB/c mice showed significantly lower levels of STUB1 expression with respect to Tregs from B6 and NOD mice (Figures 5B,C). On the other hand, USP7 expression was lower in stimulated Treg cells from BALB/c mice than in stimulated Tregs from NOD and B6 mice (Figure 5D). When STUB1/USP7 expression ratios were analyzed, significantly higher values were detected in Tregs from NOD mice at basal conditions with respect to Tregs from the other mouse strains analyzed (unstimulated, Figure 5D). Moreover, STUB1/USP7 expression ratio analyzed in Tregs stimulated with αCD3/CD28 plus IL-2, showed significantly higher values in Tregs from NOD mice with respect to Tregs from B6 and BALB/c mice (Figure 5D). In addition, NOD Tregs were stimulated with αCD3/CD28 with or without IL-2 in the absence or presence of the proteasome inhibitor MG132 (Figure 5E). Interestingly, NOD Tregs stimulated with only αCD3/CD28 in the presence of MG132 retained Foxp3 expression levels comparable to those observed in Tregs stimulated with αCD3/CD28 plus IL-2, suggesting that Foxp3 instability was related to proteasome activity (Figure 5E). Since the ubiquitin-dependent degradation of Foxp3 in Tregs can be triggered by exposure to inflammatory stress *in vitro*, we also analyzed STUB1/USP7 expression after stimulation with αCD3/CD28 and IL-2 plus IL-6 or INF-γ. As shown in Figure 5F, under stress signals, Tregs from NOD mice show higher STUB1/USP7 ratios. Indeed, in the presence of IL-6, both B6 and NOD Tregs showed higher STUB1/USP7 ratios with respect to Tregs from BALB/c mice. With the addition of INF-γ, NOD Tregs showed a significantly increased STUB1/USP7 expression ratio than Tregs from the other two mouse strains (Figure 5F). Interestingly, under some stressful conditions (abundance of IL-6), the STUB1/USP7 ratio showed no differences between Tregs from NOD and B6, suggesting once again that highly and mildly autoimmune-prone mouse strains may share some Tregs features. Altogether, these results suggest that the high expression levels of STUB1 observed in NOD Tregs could be favoring the loss of Foxp3 because of the induction of its proteasome degradation.

## Discussion

Although the IL-2/IL-2R signaling pathway has been the focus of several studies, certain aspects of its molecular regulation are still not well-characterized, and a more complete understanding of the pathway is necessary. IL-2 is essential for the maintenance of Tregs since, in its absence, a profound deficiency of Tregs occurs leading to autoimmunity (41). Studies of IL-2 and CD25 deficiency have shown that thymus generation of Foxp3^+^ Tregs may be only partially dependent on IL-2 (41–43). However, as shown by several experimental approaches, IL-2 is critical for the maintenance of functional Tregs in periphery (41–43). Certainly, it has been demonstrated that IL-2 is required for the stable expression of Foxp3 and other mediators of suppressor activity in Tregs (44).

Within 20 insulin-dependent diabetes (*Idd*) genetic regions associated with TID susceptibility and resistance, the *Idd3* region includes genes encoding IL-2 and IL-21 (45). Indeed, the NOD *Idd3* locus has been associated with reduced IL-2 expression, alterations in Treg cell pools and development of autoimmune diseases such as autoimmune diabetes, autoimmune ovarian dysgenesis, and autoimmune sialadenitis (1, 22, 24, 45–47). However, low IL-2 levels seem not to be only responsible for Tregs alterations since NOD mice containing B6-derived *Idd3* locus produce twice more IL-2 than wild type NOD but show a modest increase in the frequency of Tregs (22). In addition, when IL-2/anti-IL-2 complex therapy was used to treat NOD mice, Tregs responded poorly suggesting that both, limited IL-2 availability and defects in the IL-2R signaling, could be underlying factors involved in NOD Tregs alterations (48).

In the present work, conducting a comparative study between young NOD, B6, and BALB/c mice, we found that NOD mice showed reduced Treg cell numbers and reduced Foxp3 expression in Tregs in secondary lymphoid organs, but not in the thymus, suggesting that reduced pools of Tregs in NOD mice do not result from biased thymic differentiation. Moreover, our data demonstrated no differences between strains in Foxp3 expression in thymic Tregs, indicating that when Tregs leave the thymus they express comparable amounts of Foxp3 and that defects in maintaining Tregs numbers and Foxp3 expression levels may occur later in periphery.

It is known that when IL-2 is added to *in vitro* cultures, it reverses the anergic, non-proliferative phenotype of Tregs and promote their capacity to suppress immune responses. Results from classic Tregs functional assays performed with NOD cells reported both, Treg cell dysfunction and also T effector cell resistance to suppression, running in parallel (8, 33–35). In the present work, we evaluated Treg cell functional activation by analyzing the response of sorted Tregs after TCR ligation plus IL-2 stimulation. Even in the presence of high levels of IL-2, Tregs from NOD mice showed a lower degree of activation when compared with Tregs from the other mouse strains (B6 and BALB/c). Upon activation, Tregs from NOD mice exhibited lower upregulation of molecules associated to Treg cell function such as LAP-1, LAG-3, PD-1, PD-L1, and CD39, suggesting reduced suppressor capacity in agreement with previously reported data (6, 8, 49). Moreover, lower proliferation ability was observed in stimulated Tregs from NOD mice indicating that NOD Tregs are less able to become activated and proliferate upon stimulation with αCD3/CD28 plus IL-2, even if abundant amounts of IL-2 are available. In a similar way to that reported in *in vitro* experiments, after *in vivo* administration of IL-2/anti-IL-2 complex therapy a weak expansion of Tregs and suboptimal protection against diabetes was observed in NOD mice (48), indicating that the sole administration of IL-2 fails to completely raise Treg cell numbers in NOD mice. For most of the parameters analyzed we found major differences between NOD and BALB/c mice Tregs, while to a lesser extent between NOD and B6 Tregs. The latter may explain the controversial evidence currently reported about NOD Tregs and reinforces the fact that B6 mice are a mildly autoimmune-prone (50, 51). Interestingly, our data show that BALB/c mice have elevated counts of Tregs, which are able to be strongly activated and not so dependent on IL-2, data that could be related, at least in part, to the greater susceptibility to tumor development reported in this strain (52). In the same way, for many of the parameters analyzed, no differences were observed between Tregs from NOD and B6 mice (i.e., similar quantities, similar levels of Foxp3, CD25, PD-L1, and LAG-3 expression after stimulation), suggesting that some Tregs features are shared by highly and mildly autoimmune-prone mouse strains. These similarities in some Tregs characteristics could explain the susceptibility to the induction of several autoimmune diseases (autoimmune encephalitis, myasthenia gravis, uveitis, etc.) upon immunization with autoantigens plus adjuvants shown by B6 mice (53, 54).

Reported *in vitro* assays have shown that, in the absence of IL-2, actively proliferating Tregs loose Foxp3 expression at an accelerated rate (37, 38). Diminished maintenance of Foxp3 expression in Tregs from T1D patients have also been reported (55). Indeed, Tregs from T1D patients showed decreased pSTAT5 in response to both, high and low doses of IL-2 stimulation. Authors proposed that decreased pSTAT5 could be due to alterations in the level of expression of molecules involved in IL-2R signaling and demonstrated increased expression of the negative regulator PTNP2 in T1D patients (55). IL-2 dependent STAT5 signaling in Tregs from NOD and B6 mice was also analyzed by James et al. showing that IL-2 signaling mechanisms are functional in NOD Tregs but requires a prolonged stimulus and high IL-2 concentrations (49). In full agreement, our results indicate that, in the absence or presence of low levels of IL-2, NOD Tregs fail to maintain Foxp3 expression *in vitro*. On the contrary, most BALB/c Tregs maintained Foxp3 expression, anti-apoptotic and proliferation markers even in the absence of IL-2. The stability of Foxp3 expression has been shown to be associated with DNA hypomethylation at specific CpG-containing regions of the *foxp3* locus, in particular the CNS2 region (56), which is crucial to stabilize Foxp3 expression (57). Although in the present work we did not analyze this aspect, studies comparing the proportion of methylated CpG at the Foxp3 locus in Tregs from NOD, B6, and BALB/c mice have shown essentially identical patterns for all the mouse strains (50). That suggests that Foxp3 instability would not be consequence of epigenetic changes but rather to a lower sensibility to limited supplies of IL-2 and/or lower IL-2R signaling response.

SOCS3 is a negative feedback inhibitor that reduces signaling by IL-2R, being scarcely expressed by Tregs in contrast to the abundant expression reported in conventional T cells (58). Tregs express little or no SOCS3 protein either when freshly isolated or in response to *in vitro* stimulation (26). It has been proposed that Tregs maintain low levels of SOCS3 in situations when responsiveness to IL-2 is required, allowing a prompt response to IL-2 (26). Interestingly, SOCS3 overexpression affects the phenotype and function of Tregs (58). By using a recombinant retrovirus that allow SOCS3 protein expression, it has been shown that SOCS3-overexpressing Tregs exhibited reduced proliferation in response to TCR stimulation, being this effect even more pronounced in the presence of IL-2. In addition, SOCS3 overexpression in Tregs impaired the ability to maintain Foxp3 expression and reduced suppressive activity (58). In the present report, we showed that, upon stimulation, Tregs from NOD mice exhibited low STAT5 responses, low upregulation of functional molecules, low proliferation rates and also high levels of expression of SOCS3 mRNA suggesting that this negative feedback inhibitor could be involved in the abnormal functions reported for NOD Tregs.

It has been reported that GRAIL is highly expressed in Tregs. Moreover, its forced expression by conventional T cells favors their conversion to iTregs (28, 59). Furthermore, GRAIL-deficient mice are resistant to immune tolerance induction and exhibit greater susceptibility to autoimmune diseases than wild-type mice. Analysis of Treg cell populations in GRAIL-deficient mice showed that GRAIL-deficient Tregs displayed reduced suppressive function and increased Th17 cell-related gene expression (59). Due to its known ubiquitin ligase activity, it would be interesting to know all the putative protein substrates of GRAIL. It has been demonstrated that GRAIL is involved in the degradation of the TCR-CD3 complex through the ubiquitin-dependent proteasome degradation pathway. Indeed, by targeting TCR-CD3 for degradation, GRAIL restricts NFATc1 expression in Tregs and naïve T cells (59). Additionally, it has been shown that GRAIL regulates the expression of the costimulatory molecule CD40L on CD4 T cells by ubiquitination (60). Interestingly, CD40/CD40L interaction it has been reported that regulates CD4^+^CD25^+^ Treg cell homeostasis (61). Furthermore, it has been reported that GRAIL ubiquitinates and degrades CD83 on CD4 T cells, a molecule which expression on Tregs is essential for their differentiation upon activation (62, 63). Curiously, it has been suggested that GRAIL could affect SOCS3 E3 ligase activity, thereby obstructing pJAK1 degradation. pJAK1 degradation is normally accomplished by the formation of SOCS3 multiunit ubiquitin “cullin ring ligase” complexes, thus stopping pSTAT5 mediated transcription and allowing prolonged transcription of Tregs centric genes under the control of STAT5b (64, 65). In Tregs, the signaling cascade downstream IL-2R permitted markedly prolonged activity of pSTAT5. It is possible that GRAIL's presence in Tregs would allow prolonged activation of pJAK1 by preventing SOCS3 E3 ligase activity that normally degrades pJAK1 (66). In the present work, we report lower mRNA and protein levels of GRAIL in Tregs from NOD mice with respect to B6 and BALB/c mice. In addition, we report enhanced levels of the GRAIL's regulator OTUB1, suggesting that a reduced GRAIL activity could be impairing IL-2R signaling. To our knowledge, this is the first report not only describing the expression levels of GRAIL and OTUB1 in Tregs from different mouse strains, but also showing that Tregs from autoimmune-prone mice have diminished levels of GRAIL. Interestingly, reduced GRAIL and Cbl-b levels have been reported in CD4^+^ T cells from lupus patients, a defect that correlated with reduced numbers of Tregs in periphery (67).

Finally, the levels of STUB-1 and USP7, enzymes reported to affect Foxp3 cellular deposits in Tregs (14, 39, 40, 68), were also evaluated in the present study. Our results showed enhanced levels of the ubiquitination enzyme STUB-1 and diminished levels of the deubiquitinating protease USP7 in Tregs from NOD mice when compared with B6 and BALB/c mice. We also observed enhanced STUB1/USP7 ratios in NOD Tregs both, under TCR plus IL-2 stimulation and also under inflammatory stress signals. Remarkably, in the presence of a proteasome inhibitor, Tregs from NOD mice were able to retain Foxp3 expression to levels comparable to those observed in conditions of TCR stimulation plus high doses of IL-2, suggesting the proteasome participation in Foxp3 instability. On the one hand, it has been described that USP7 in Tregs is capable of interacting with Foxp3 to catalyze its deubiquitination (40, 69). Moreover, van Loosdregt et al. have shown that knocking down USP7 reduces Foxp3 levels in Tregs and inhibits its functions (40). These authors have also shown that the overexpression of USP7 in Tregs increases Foxp3 protein levels (40). Besides, the conditional deletion of USP7 in Tregs leads to a dramatic loss of immune regulation and to lethal autoimmunity, indicating the need to counteract the process of Foxp3 ubiquitination in order to maintain a functional and stable pool of Tregs in periphery (14). In agreement with all these reported data, our results suggest that individuals in whom lower Tregs USP7 activity is observed would be those in which lower quantities of Tregs and lower Foxp3 expression are found. On the other hand, ectopic STUB1-mediated Foxp3 loss has been shown to disrupt the suppressive function of Tregs, reduce the expression of Tregs-associated genes, and upregulate the expression of effector-type cytokines, such INF-γ (39, 70). Conversely, knocking down STUB1 stabilizes Foxp3 expression and increases the suppressive potency of Tregs (39). Herein, we provide data indicating that Tregs from the autoimmune-prone NOD mice have a significantly lower ability to retain Foxp3 expression with respect to Tregs from B6 and BALB/c mice. In addition, we report that NOD Tregs have significantly higher STUB1/USP7 expression ratios than B6 and BALB/c Tregs, suggesting that an unfavorable balance in ubiquitination factors, together with an altered IL-2R signaling, could be factors underlying the functional defects shown by Tregs from NOD mice.

Restoring the immune balance through the amplification of Tregs represents a promising strategy to treat autoimmune diseases. However, Foxp3^**+**^ Tregs might become unstable; so, therapies combining the transfer of Tregs with Tregs-stabilizing drugs are expected to be the most effective ones to restrain autoimmune diseases. To select the appropriate stabilizing drug, it is important to know the exact molecular targets and cellular pathways that are particularly affected in autoimmune prone individuals. Our results show the differential expression of SOCS3, GRAIL, and STUB1/USP7 in Tregs from the autoimmune-prone NOD mice, factors that may affect IL-2R signaling and Foxp3 stability, resulting in insufficient and/or defective Tregs to maintain self-tolerance. We envision that these results may have significant implications for translational research in human diseases.

## Data Availability Statement

The datasets generated for this study are available on request to the corresponding author.

## Ethics Statement

The animal study was reviewed and approved by Committee for Animal Care and Use of the Facultad de Ciencias Químicas, Universidad Nacional de Córdoba (Res. HCD 240/216).

## Author Contributions

VR and RM obtained funding, conceived and designed the experiments. GG, CO, DP, and FS performed the experiments. VR and GG analyzed the data and achieved data interpretation and drafting. YA and CS contributed by providing reagents, materials, and analysis tools. VR, RM, GG, and CS contributed to the writing of the manuscript. All authors have contributed to the revision of the work, provided important intellectual content, and carefully reviewed and approved the final version of the manuscript. Authors agree to be accountable for all aspects of the work in terms of accuracy or integrity and other related aspects.

### Conflict of Interest

The authors declare that the research was conducted in the absence of any commercial or financial relationships that could be construed as a potential conflict of interest.
