# Supplementary material for: T Regulatory Cells From Non-obese Diabetic Mice Show Low Responsiveness to IL-2 Stimulation and Exhibit Differential Expression of Anergy-Related and Ubiquitination Factors
Source: Front Immunol. 2019 Nov 25;10:2665. doi: 10.3389/fimmu.2019.02665 (PMC6886461; doi:10.3389/fimmu.2019.02665)
Supplement: Supplementary file 1 [file Data_Sheet_1.pdf]

*Supplementary Material*

**Supplementary Figure 1:** *Frequencies of $CD4^{+}CD25^{+}Foxp3^{+}$ Treg cells in lymphoid organs from NOD, B6, and BALB/c mice.*

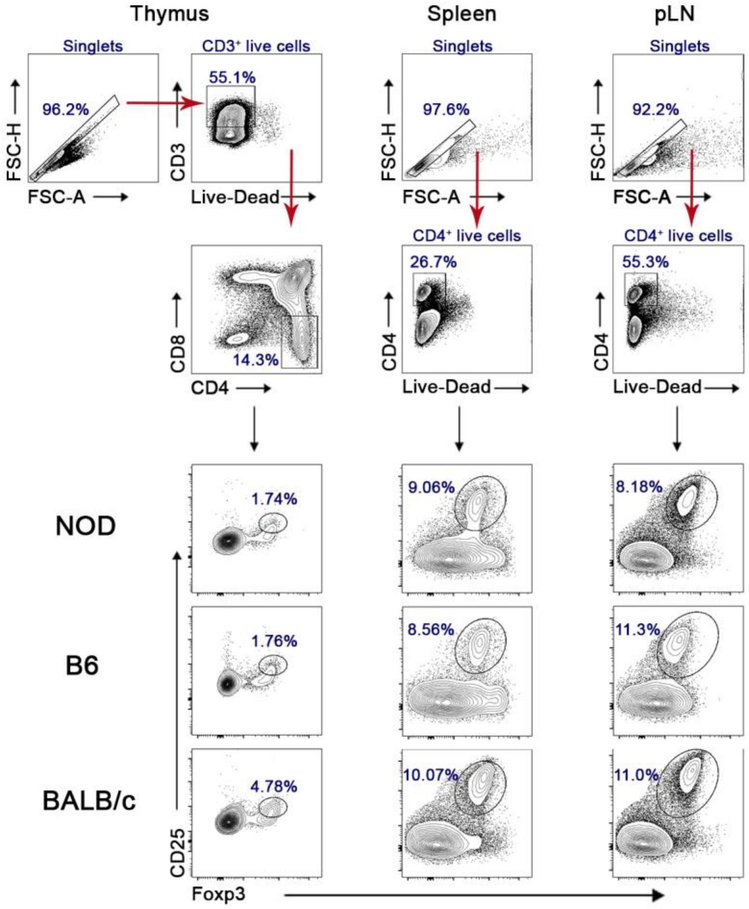


(A) Representative flow cytometry plots showing the gating strategy to identify Treg cells in lymphoid organs from NOD, B6, and BALB/c mice (top). Representative contour plots of the frequencies values of $CD25^{+}Foxp3^{+}$ cells are shown for each mouse strain (bottom).

**Supplementary Figure 2:** *Gating strategy used to identify CD4$^{+}$Foxp3$^{+}$ cells in sorted Tregs from NOD, B6, and BALB/c mice.*

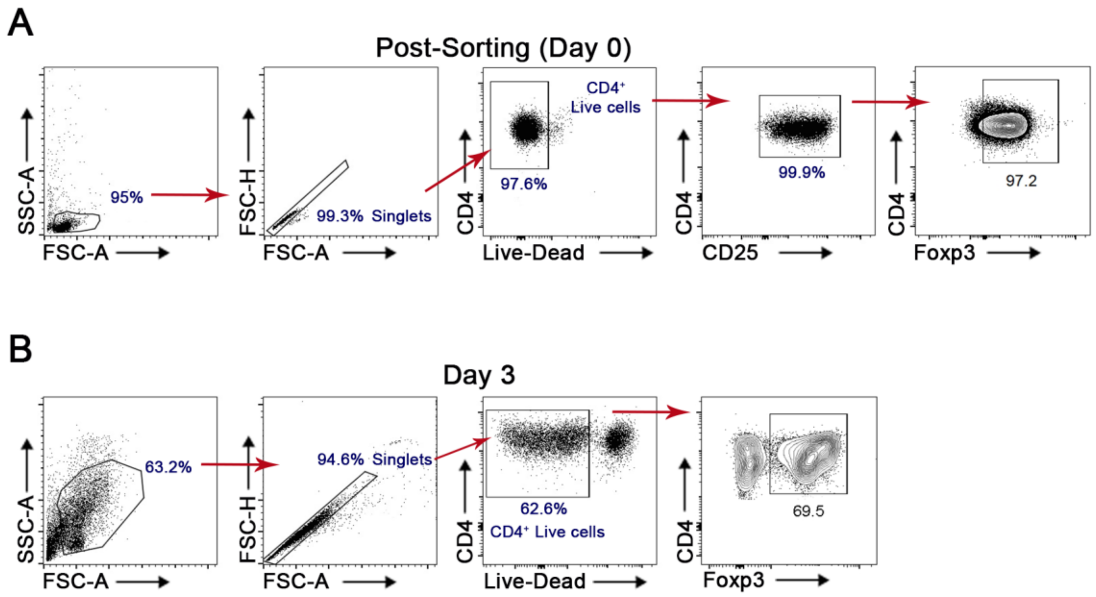


Representative flow cytometry plots showing the gating strategy used to identify Foxp3$^{+}$ Treg cells after cell sorting isolation (A) or 3 days of αCD3/CD28 stimulation in the presence or absence of rIL-2 (B).
